# *In Situ* Molecular Ecological Analyses Illuminate Distinct Factors Regulating Formation and Demise of a Harmful Dinoflagellate Bloom

**DOI:** 10.1128/spectrum.05157-22

**Published:** 2023-04-19

**Authors:** Liying Yu, Tangcheng Li, Hongfei Li, Minglei Ma, Ling Li, Senjie Lin

**Affiliations:** a State Key Laboratory of Marine Environmental Science and College of Ocean and Earth Sciences, Xiamen University, Xiamen, China; b Central Laboratory, Second Affiliated Hospital of Fujian Medical University, Quanzhou, China; c Department of Marine Sciences, University of Connecticut, Groton, Connecticut, USA; Swansea University

**Keywords:** *Karenia longicanalis*, metatranscriptome, DNA barcoding, HAB, microeukaryotes, prokaryotic microbes, algae, dinoflagellate

## Abstract

The development and demise of a harmful algal bloom (HAB) are generally regulated by multiple processes; identifying specific critical drivers for a specific bloom is important yet challenging. Here, we conducted a whole-assemblage molecular ecological study on a dinoflagellate bloom to address the hypothesis that energy and nutrient acquisition, defense against grazing and microbial attacks, and sexual reproduction are critical to the rise and demise of the bloom. Microscopic and molecular analyses identified the bloom-causing species as *Karenia longicanalis* and showed that the ciliate *Strombidinopsis* sp. was dominant in a nonbloom plankton community, whereas the diatom *Chaetoceros* sp. dominated the after-bloom community, along with remarkable shifts in the community structure for both eukaryotes and prokaryotes. Metatranscriptomic analysis indicated that heightened energy and nutrient acquisition in *K. longicanalis* significantly contributed to bloom development. In contrast, active grazing by the ciliate *Strombidinopsis* sp. and attacks by algicidal bacteria (*Rhodobacteracea*, *Cryomorphaceae*, and *Rhodobacteracea*) and viruses prevented (at nonbloom stage) or collapsed the bloom (in after-bloom stage). Additionally, nutrition competition by the *Chaetoceros* diatoms plausibly contributed to bloom demise. The findings suggest the importance of energy and nutrients in promoting this *K. longicanalis* bloom and the failure of antimicrobial defense and competition of diatoms as the major bloom suppressor and terminator. This study provides novel insights into bloom-regulating mechanisms and the first transcriptomic data set of *K. longicanalis*, which will be a valuable resource and essential foundation for further elucidation of bloom regulators of this and related species of Kareniaceae in the future.

**IMPORTANCE** HABs have increasingly occurred and impacted human health, aquatic ecosystems, and coastal economies. Despite great efforts, the factors that drive the development and termination of a bloom are poorly understood, largely due to inadequate *in situ* data about the physiology and metabolism of the causal species and the community. Using an integrative molecular ecological approach, we determined that heightened energy and nutrient acquisition promoted the bloom, while resource allocation in defense and failure to defend against grazing and microbial attacks likely prevented or terminated the bloom. Our findings reveal the differential roles of multiple abiotic and biotic environmental factors in driving the formation or demise of a toxic dinoflagellate bloom, suggesting the importance of a balanced biodiverse ecosystem in preventing a dinoflagellate bloom. The study also demonstrates the power of whole-assemblage metatranscriptomics coupled to DNA barcoding in illuminating plankton ecological processes and the underlying species and functional diversities.

## INTRODUCTION

The aquatic ecosystem is threatened by harmful algal blooms (HABs), which have increased in recent decades in frequency, bloom species diversity, geographic extent, and severity of ecological and economic damages (1, 2). HABs result from the rapid growth of one or few microalgae due to natural processes (such as upwelling relaxation) or eutrophication from anthropogenic loadings (3, 4). However, algal bloom dynamics are regulated by cell division rate (μ), current-mediated cell-concentrating (import or I) or -dissipating (export or Ex) factors, grazing by animals (G), and mortality (M) due to environmental stress and microbial attacks (algicidal bacteria and viruses). These factors can be conceptually expressed as follows: d*N*/d*t* = μ + I − Ex − G − M, where *N* and *t* are population abundance and time period, respectively. Some blooms are believed to be initiated from seed populations brought onshore by currents (5) or dissipated by rain and currents (6). Currents also can have important indirect effects by bringing in nutrients. However, most HABs result from the dynamics of μ, G, and M, whereas I or Ex has minimal direct effects. To date, studies on the regulators of HABs have mainly focused on abiotic factors that regulate μ, primarily water temperature and nutrient conditions (7–10). In contrast, biotic interactions reflected in G and M, which are also crucial regulators of bloom development and decline, have been less studied. Among the associated microbes, bacteria are a major constituent, and up to 20% of them are attached to algae or particles (11). The main effects of microbes (bacteria and viruses) on HABs include the lysis of algal cells (12–14) and inhibition of algal growth (15), although some bacteria can promote algal growth (16–18). It is highly challenging to determine which of the multiple growth-promoting variables (e.g., light, nutrients) and bloom-depressing variables (e.g., grazing, microbial attacks) are the drivers of bloom emergence and decline, because this requires simultaneous and *in situ* measurements at the species level. An integrative molecular ecological approach, however, makes such an endeavor possible, since it allows investigators to analyze field samples directly to reconstruct the metabolic profile of the bloom species *in situ*, in comparison to that of other organisms in the same habitat, in the course of bloom evolution.

Metabarcoding and metatranscriptome profiling with next-generation sequencing have proven to be effective ways to profile community composition and metabolic landscape (19, 20) and to detect major shifts in metabolic activities and resource utilization in the course of phytoplankton succession (21, 22). Metatranscriptomic studies have successfully tracked cellular processes associated with life history transitions, nutrient acquisition, defense, and energy production during dinoflagellate blooms by community gene expression analyses (23–30). Previous data also suggested that sexual reproduction (meiosis) could contribute to bloom formation for dinoflagellate blooms (25, 31). How grazing by zooplankton and attacks by microbes influence the bloom dynamics were not addressed in those studies.

Marine HAB causative species are predominantly dinoflagellates (32–36). Species of the genus *Karenia* are frequent bloom-forming dinoflagellates in coastal waters (37–40). Most *Karenia* species, such as *Karenia brevis* and *Karenia mikimotoi*, produce toxins that directly kill marine organisms, causing immense economic losses and environmental damages (19, 41). Extensive research has been conducted to investigate the ecological drivers (mainly nutrients and other environmental factors) of *Karenia* blooms (42–45). For example, a study suggested that N, P, and Si nutrients are important divers of a *K. mikimotoi* bloom (44). Similarly, inorganic and organic N and P nutrients have been shown to be important to *K. brevis* bloom outbreaks (46). However, due to the complexity of interactions between the species (genetics) and environmental (abiotic and biotic) factors in each bloom, it is hard to generalize the findings, and each bloom's precise driver and terminator are still poorly understood.

In this study, we investigated a *Karenia* bloom that occurred in Tongxin Bay of Fujian, China, in June 2018 using an integrative molecular ecological approach. We collected samples from the bloom, nonbloom (as a surrogate of prebloom), and after-bloom conditions within the bay. Metabarcoding analyses based on small subunit (SSU) ribosomal DNA (rDNA; 16S and 18S) were performed to profile algal and bacterial community structures and their changes across the different bloom conditions. In parallel, whole-assemblage metatranscriptomic analyses were carried out to identify critical metabolic processes associated with the different bloom conditions. This research portrays prokaryotic and eukaryotic community shifts among the bloom, nonbloom, and after-bloom stages and sheds light on what main biotic factors and metabolic processes associated with energy and nutrient acquisition, cell proliferation, and defense drive the bloom development and demise in a seriously understudied dinoflagellate.

## RESULTS

### Bloom profile and plankton community shift through bloom stages.

**FIG 1:**
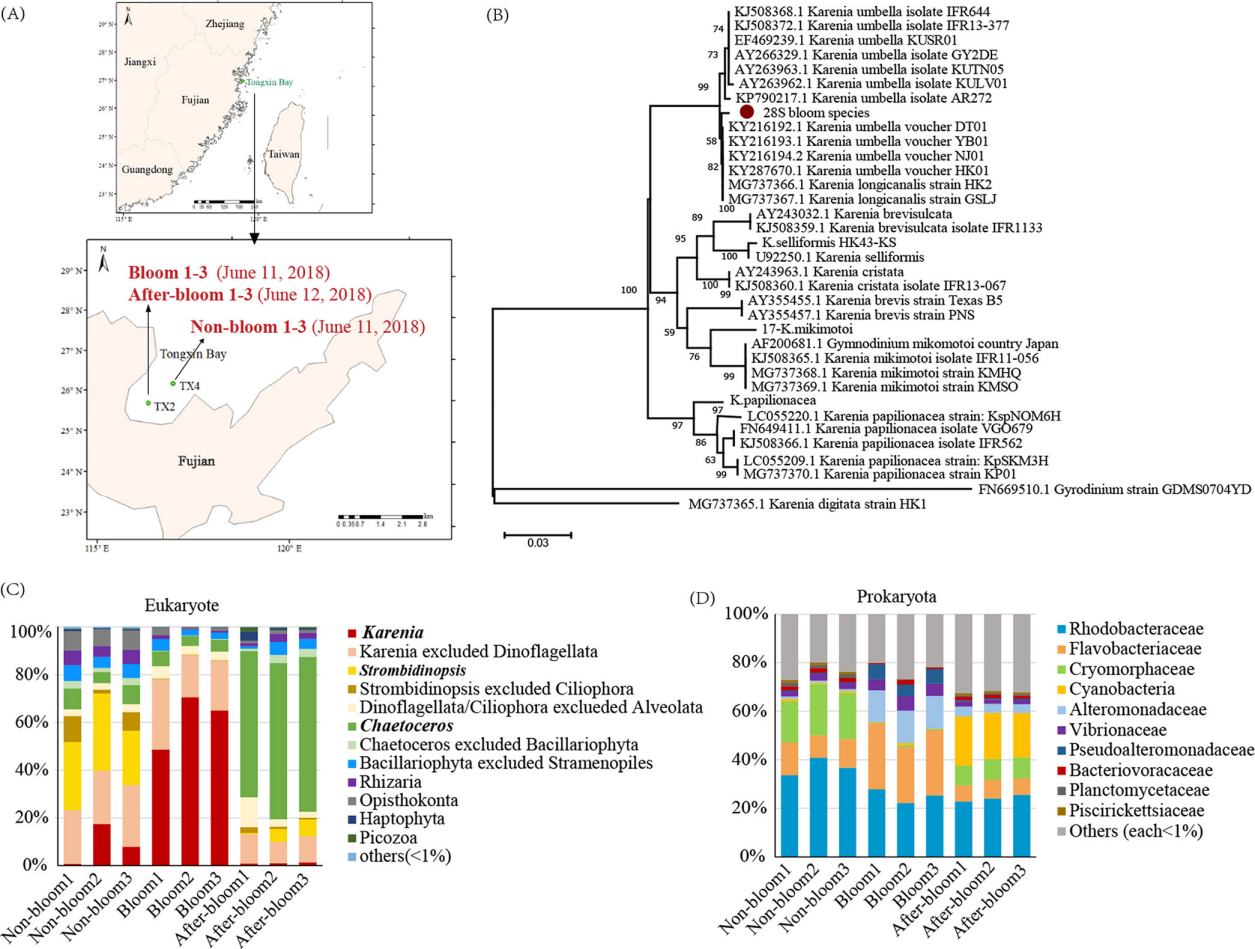
Study site and bloom community profile. (A) Map of study site and sampling stations and dates. (B) Maximum-likelihood phylogenetic analysis of 28S rDNA sequences showing that the bloom species is *Karenia longicanalis* (= *K. umbella*). Scale bar represents number of substitutions per site. (C) Eukaryotic plankton community composition showing *Karenia* bloom (red) development and demise. (D) Prokaryotic plankton community composition showing shifts of dominant bacterial lineages throughout different stages of the bloom.

At the community level, the chlorophyll *a* concentration was 16.8 μg/liter in the bloom samples, compared with 1.1 μg/liter in the nonbloom samples and 1.5 μg/liter in the after-bloom samples (Fig. 1A), indicating a >10-fold-higher phytoplankton community biomass in the bloom than the nonbloom and after-bloom conditions, the latter two of which showed similarly low phytoplankton community biomass. Microscopic observations and molecular analysis (28S rDNA) indicated that the bloom species was *K. longicanalis* (formerly *K. umbella* [47]) (Fig. 1B), and its abundance was 1.51 × 10^7^ cells/liter in the bloom samples, compared to 2.4 × 10^4^ cells/liter (a 600-fold-lower concentration) in the nonbloom samples, and it declined by 137-fold (1.1 × 10^5^ cells/liter) in the after-bloom samples. Based on 18S rDNA metabarcoding of the community (Fig. 1C), *Karenia* sp. accounted for 8.71%, 61.42%, and 1.09% in the nonbloom, bloom, and after-bloom groups of samples, respectively.

The 18S rDNA metabarcoding analysis also showed that the nonbloom plankton community was dominated by the ciliate *Strombidinopsis* sp. (accounting for 27.87%), and the after-bloom community was dominated by the diatom *Chaetoceros* sp. (accounting for 63.86%) (Fig. 1C). Other major eukaryotic phyla included Opisthokonta (0.58% to 8.20%), Rhizaria (0.36% to 6.25%), Haptophyta (0.16% to 3.94%), and *Picozoa* (0.10% to 1.75%) throughout this study period (Fig. 1C).

To explore what algae-associated microbes might be involved in the bloom development and decline, the microbial community was analyzed using 16S rDNA metabarcoding. Ten dominant families of bacteria were identified (Fig. 1D): Rhodobacteraceae (22.29% to 40.81%), Flavobacteriaceae (6.72% to 27.28%), Cryomorphaceae (0.34% to 21.22%), Cyanobacteria (0.04% to 20.40%), Alteromonadaceae (8.71% to 13.54%), Vibrionaceae (1.72% to 5.86%), Pseudoalteromonadaceae (0.31% to 6.10%), Bacteriovoracaceae (0.43% to 2.05%), Planctomycetaceae (0.06% to 1.47%), and Rickettsiaceae (0.01% to 1.17%). Rhodobacteracea were consistently abundant across sample groups. Flavobacteriaceae, Alteromonadaceae, Vibrionaceae, and Pseudoalteromonadaceae had a higher relative abundance in the bloom sample group than in the nonbloom and after-bloom sample groups. Cryomorphaceae showed the highest relative abundance in the nonbloom sample group, whereas Cyanobacteria were most abundant in the after-bloom sample group.

### Environmental and biotic factors showing correlations with the abundance of bloom species.

**FIG 2:**
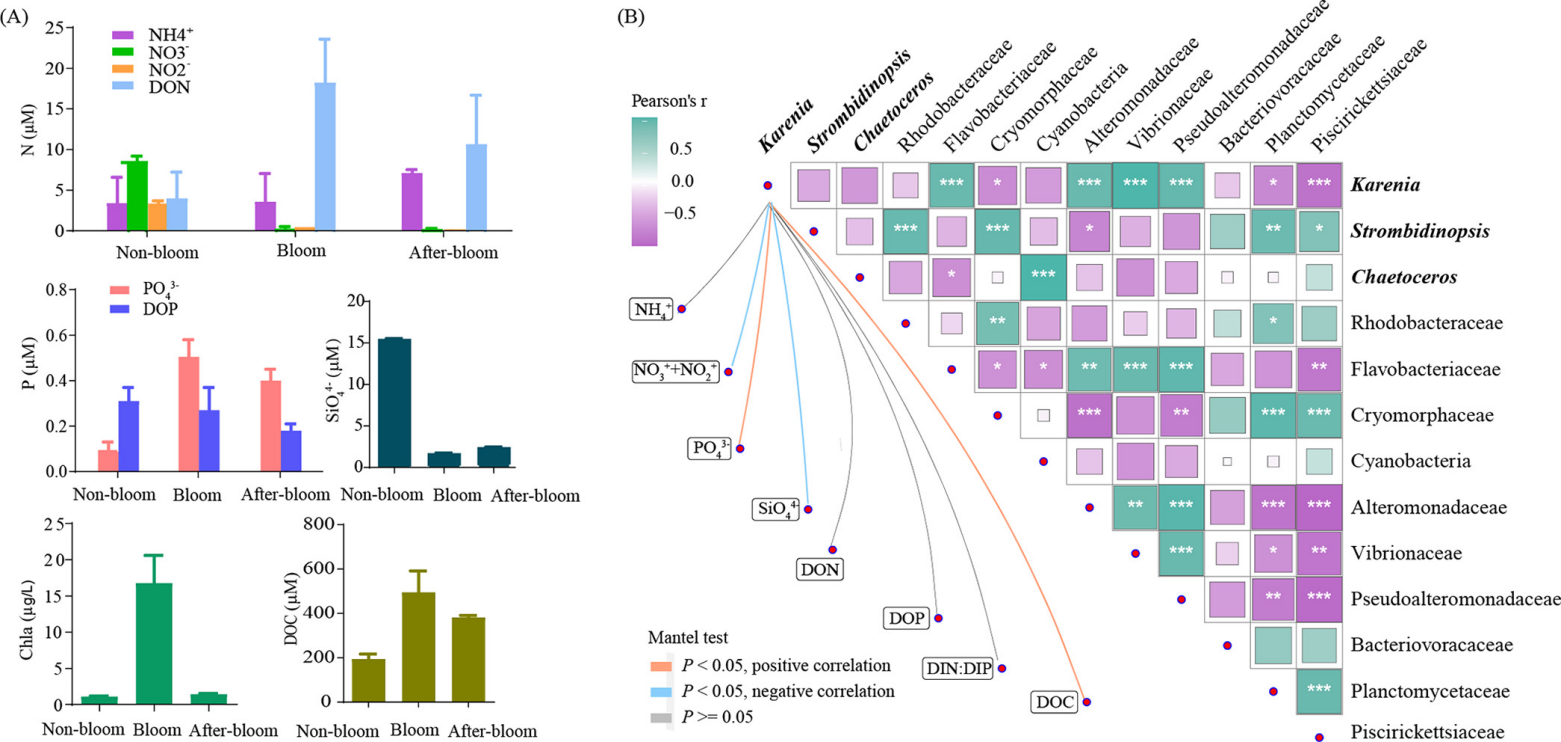
Quantitative relationships of lineages within the community with nutrient variables. (A) Changes of nutrient concentrations, chlorophyll, and dissolved organic carbon concentrations with bloom conditions. (B) Correlations of dominant lineages with each other and with nutrient conditions. Correlations between lineages (filled squares) were analyzed using the Pearson test. Deeper color and larger squares mark stronger correlations. *, *P* = 0.01 to 0.05; **, *P* < 0.01; ***, *P* < 0.001. Correlations between *Karenia* and nutrient factors (connecting lines) were analyzed using the Mantel test. The orange and blue connecting lines mark positive and negative correlations between the relative abundance levels of *Karenia* and nutrient concentrations.

The relative abundance of *Karenia* (based on 18S metacoding) was negatively correlated with NO_3_^−^, NO_2_^−^, and SiO_4_^3−^ but positively correlated with PO_4_^3−^ and dissolved organic carbon (DOC) concentrations (Fig. 2B). Besides, the *Karenia*-dominated bloom samples displayed the highest dissolved oxygen, pH, and surface irradiance in the environment (see Table S1 in the supplemental material), indicating active photosynthesis. The Pearson test showed that the relative abundance of *Karenia* was positively correlated with the abundances of bacteria from the families Flavobacteriaceae, Alteromonadaceae, Vibrionaceae, and Pseudoalteromonadaceae but negatively correlated with that of Cryomorphaceae, Planctomycetaceae, and Piscirikettsiaceae (Fig. 2B), suggesting potential promoting and depressing effects on the bloom, respectively.

### Community metatranscriptomic profile and its shift through bloom stages.

Our whole-assemblage metatranscriptome sequencing yielded a total of over 1.15 billion cDNA reads (Table S2). After quality filtering, the resulting 0.91 billion (79.11%) clean reads were assembled into a reference metatranscriptome composed of 1,389,260 unigenes with an *N*_50_ of 868 bp and a maximum length of 131,207 bp (Table S3). The reference metatranscriptome showed a total read mapping rate of 77.63 to 90.88% (Table S4), indicating an overall good assembly quality. Functional annotation based on the eggNOG, Swiss-Prot, KEGG, GO, and nrMegPhylodb databases gave a total annotation rate (genes annotated by at least one of these databases) of 74.87% (Table S5).

**FIG 3:**
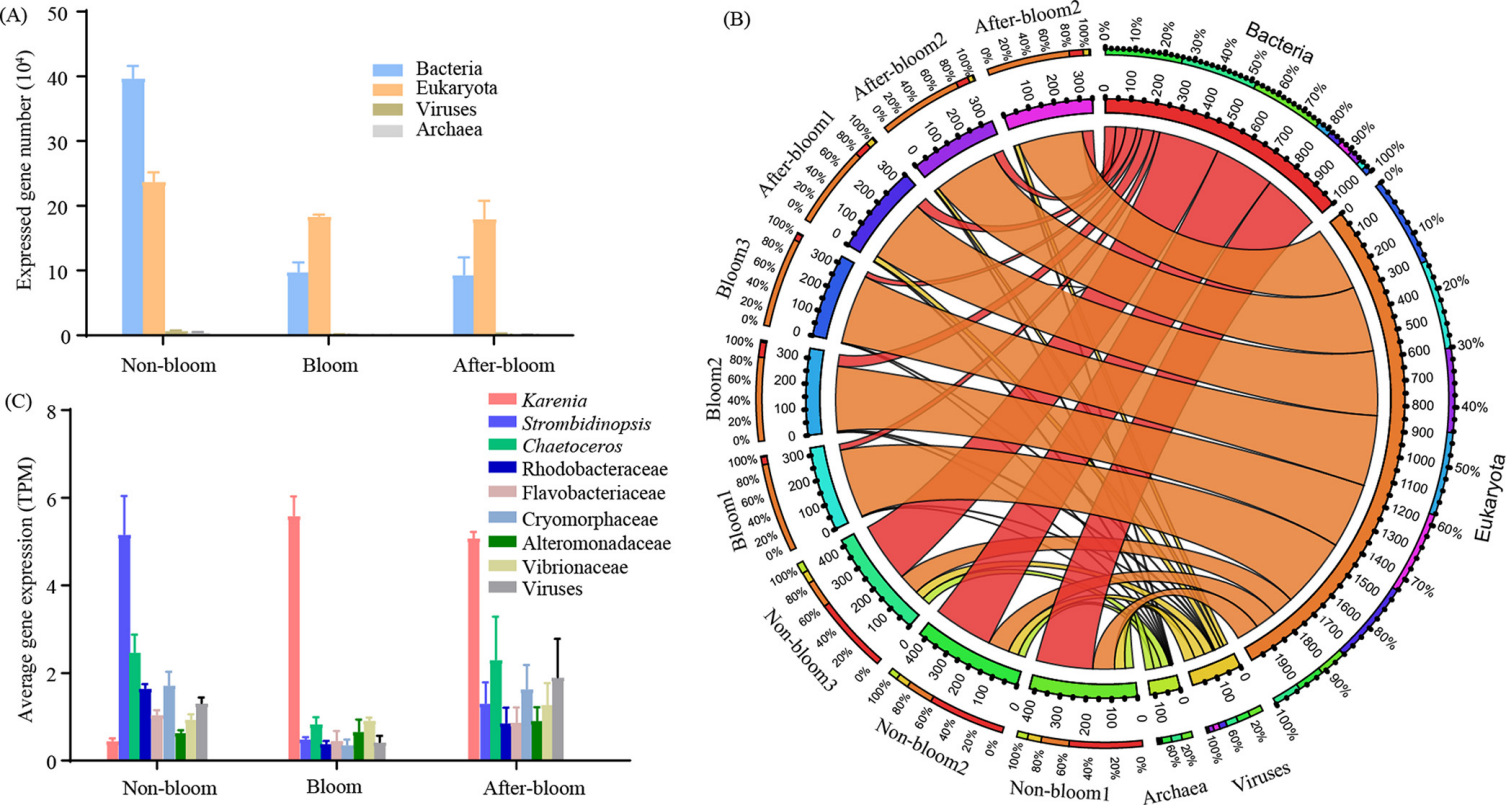
Community metabolic profiles based on community TPM in the three bloom stages. (A) Expressed gene diversity in the four kingdoms. The average gene diversity of three biological replicates is displayed for each group. (B) Circos plot summarizing gene expression (TPM) contribution of the four kingdoms to each sample. The different colors of the inner ring mark total TPM of different samples or kingdoms. The outer ring shows the gene expression percentages of kingdoms in samples or gene expression percentages of samples in kingdoms, with colors corresponding to the inner ring. Wider connecting ribbons between kingdom and sample indicate a larger gene expression contribution of this kingdom in the sample. (C) Average gene expression level (total TPM divided by number of genes with read count of >0) in the dominant lineages.

Among the 1,389,260 unigenes, 74.32% were assigned taxa, of which 58.20% were from bacteria, 40.04% from eukaryotes, 1.08% from viruses, and 0.69% from archaea. The number of expressed genes and the expression levels of the genes from bacteria, viruses, and archaea decreased markedly from the nonbloom samples to the bloom samples (Fig. 3A). Gene expression (transcript per million mapped reads [TPM]) of the bloom and the after-bloom assemblages were dominated by eukaryotes (about 80%), whereas that of the nonbloom samples were dominated by bacteria (~60%) followed by ~20% eukaryotes (Fig. 3B). At the family or genus level, however, the bloom metatranscriptomes were dominantly contributed by *Karenia*, while the nonbloom metatranscriptomes were contributed mainly by the ciliate *Strombidinopsis*, the diatom *Chaetoceros*, the bacteria *Crymorphaceae* and *Rhodobacteracea*, and viruses, in decreasing order. The after-bloom metatranscriptomes were mainly composed of transcripts from *Karenia*, *Chaetoceros*, viruses, Crymorphaceae, and *Strombidinopsis* (Fig. 3C). The gene expression levels of *Strombidinopsis* in the bloom sample group were >20-fold lower than in the nonbloom sample group. Moreover, *Chaetoceros*, Rhodobacteracea, Crymorphaceae, and virus gene expression levels were much higher (>3-fold) in the nonbloom samples than in the bloom samples.

### *K. longicanalis* transcriptome and bloom-associated differential expression of genes related to energy and nutrient acquisition, defense, and cell division cycle (ENDS).

**FIG 4:**
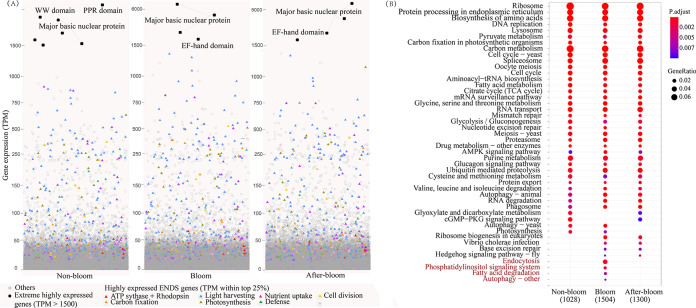
Abundantly expressed genes in *Karenia* under the three bloom conditions (nonbloom, bloom, and after-bloom). (A) Extreme highly expressed genes (TPM of >1,500; black dot) and highly expressed (top 25% expressed genes; colored triangles) ENDS genes in *Karenia*. (B) KEGG enrichment of highly expressed genes (top 25% expressed genes). The pathway in red font represents those with DEGs uniquely enriched under the bloom condition.

As this is the first transcriptome ever reported for *K. longicanalis*, particularly during a natural bloom, it is of interest to catalog the expressed genes. From the metatranscriptomes, 94,785 unigenes were identified as *Karenia* genes. Four genes were expressed constantly at >1,500 TPM across all sample groups, including three major basic nuclear protein genes and one EF-hand domain-containing protein gene (Fig. 4A). Using a TPM of the >75% quartile as the criterion of highly expressed genes (HEGs), we found 9,021, 17,533, and 13,623 HEGs of *Karenia* in the nonbloom, bloom, and after-bloom samples, respectively. KEGG enrichment of the HEGs revealed that 33 pathways were commonly enriched in all three sample groups, such as the ribosome, protein processing in the endoplasmic reticulum, and biosynthesis of amino acids (Fig. 4B). Besides, we found that HEGs of endocytosis, phosphatidylinositol signaling system, fatty acid degradation, and autophagy, representing pathways of mixotrophic uptake and recycling of nutrients, energy metabolism, and signaling, were uniquely enriched in the bloom sample group (Fig. 4B).

**FIG 5:**
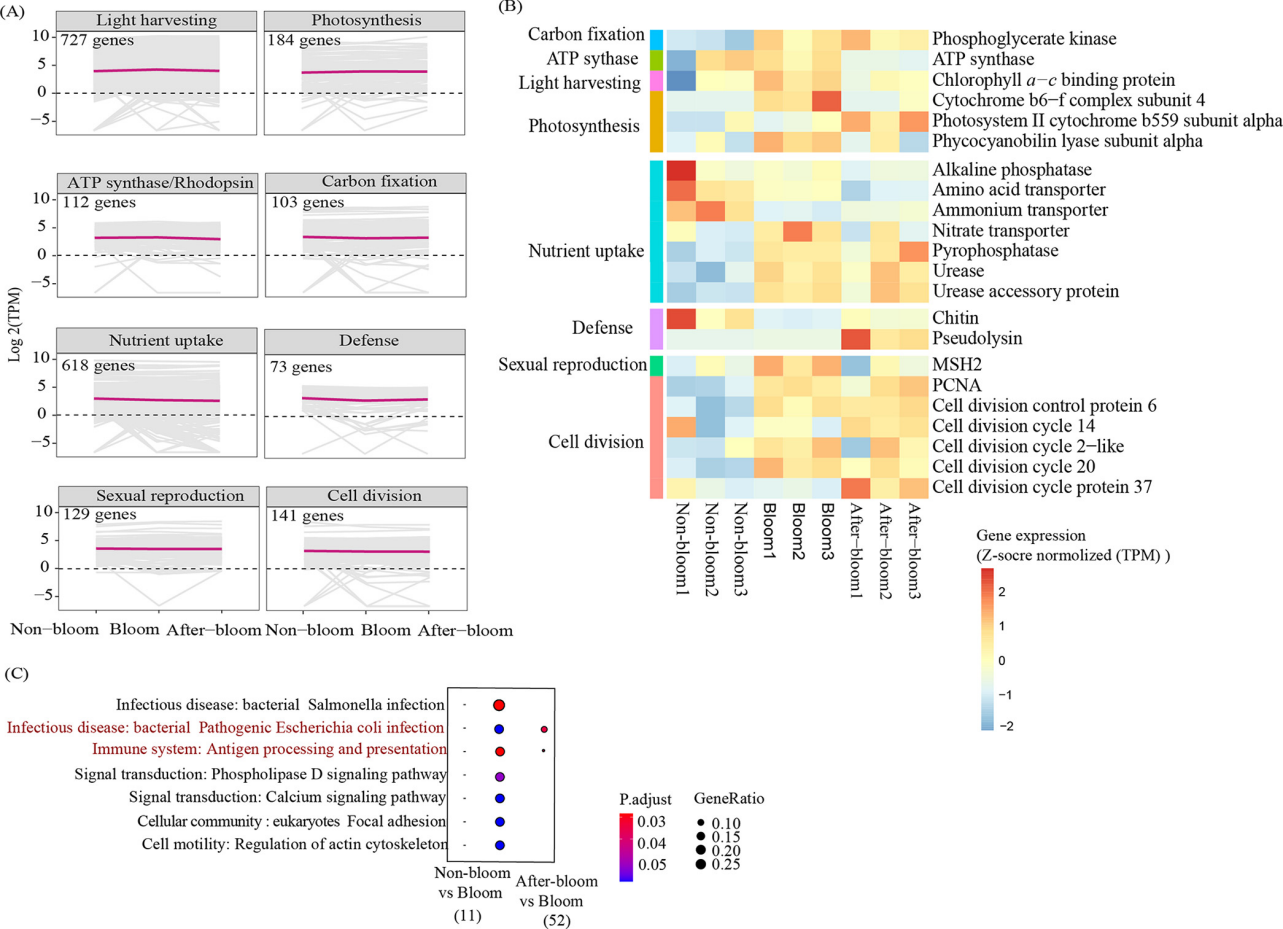
Energy and nutrient acquisition, defense, and cell reproduction (ENDS)-related genes and differentially expressed genes in *Karenia* under the three bloom conditions (nonbloom, bloom, and after-bloom). (A) Expression patterns of ENDS genes in *Karenia*. (B) Differentially expressed ENDS genes in the bloom stage compared to nonbloom and after-bloom stages. Differentially expressed genes were defined as those with *P* values of <0.05 using the *t* test. (C) KEGG enrichment of differentially expressed genes (adjusted *P* value of <0.05 and fold change of ≥2) in the bloom stage compared to nonbloom and after-bloom stages. The pathway in red font represents common enriched pathways in both bloom versus nonbloom and bloom versus after-bloom comparisons by DEGs upregulated in the bloom condition.

To examine how the capacity of ENDS genes influenced the bloom dynamics, we documented the features of related genes or pathways associated with the bloom. In total, we detected 1,126 energy acquisition genes (E) involved in light harvesting, photosynthesis, proton pump rhodopsin, and carbon fixation and genes of ATP synthase; 618 nutrient uptake genes (N), such as N-, P-, and Si-related transporters; 73 defense genes (D); and 270 cell proliferation genes comprising those regulating sexual reproduction (meiosis) and cell division cycle (S) in *Karenia* (Fig. 5A; Fig. S1). These ENDS genes were relatively stable across the three samples (Fig. 5A). Among the *Karenia* 2,087 ENDS genes identified, 891 (42.69%), 1,001 (47.96%), and 897 (42.98%) were highly expressed in nonbloom, bloom, and after-bloom samples, at higher percentages than the overall percentage of HEGs (25%) (Fig. 4A).

Despite the overall stable high expression of ENDS genes, we observed some slight changes. There were 22 differentially expressed ENDS genes (Fig. 5B). Among them, most of the ENS genes were upregulated, but defense genes were downregulated in the bloom samples compared to the nonbloom or after-bloom samples. Energy metabolism-related differentially expressed genes (DEGs) that were upregulated during bloom included ATP synthase and genes involved in light harvesting (chlorophyll *a* to *c* binding protein gene family), photosynthesis, and carbon fixation. Nutrient uptake-related upregulated DEGs included amino acid transporter, nitrate transporter, pyrophosphatase, urease and urease accessory protein. Related to cell proliferation were DEGs of the cell cycle proteins and MSH2, which were upregulated in the bloom (Fig. 5B). Regarding defense (D), the chitin and pseudolysin genes annotated as encoding antimicrobial peptides were significantly downregulated in bloom samples relative to the nonbloom or after-bloom sample groups (Fig. 5B). Additionally, we detected 3,627 total DEGs between the bloom and nonbloom sample groups, but only 257 DEGs between the bloom and after-bloom sample groups, indicating more similar transcriptomic status between the bloom and after-bloom stages. KEGG enrichment results showed that the upregulated DEGs in the nonbloom samples were enriched in defense-related pathways against two bacteria-mediated infectious diseases and a pathway related to the immune system. Two of the three defense-related pathways were also upregulated in the after-bloom samples (Fig. 5C).

### Differential nutrient uptake strategies between *Karenia* bloom and *Chaetoceros* dominance.

**FIG 6:**
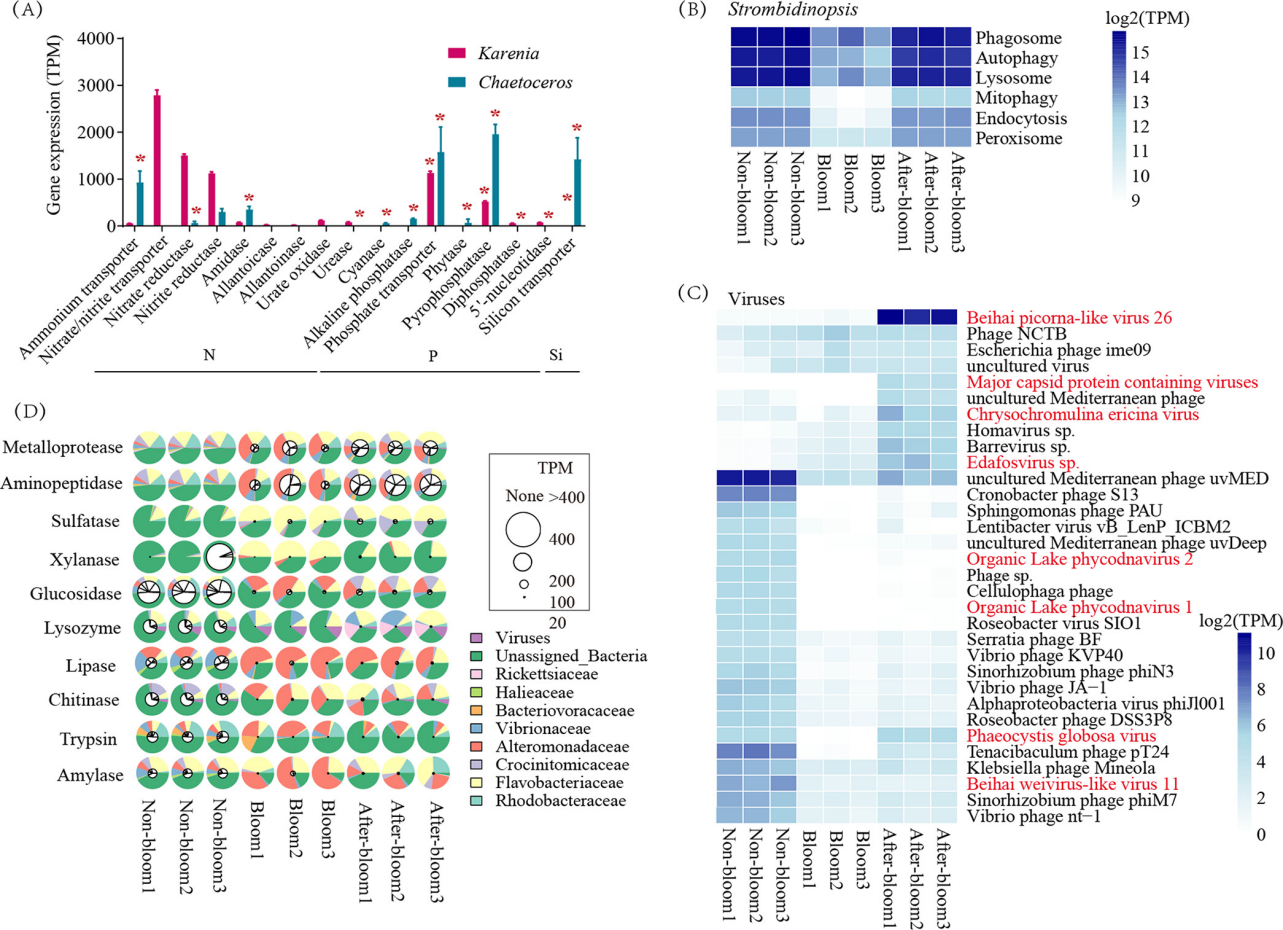
Expression of genes in the dominant species of phytoplankton, ciliate *Strombidinopsis*, viruses, and prokaryotes at the three bloom stages. (A) Highly expressed genes (top 25% expressed genes) related to nitrogen (N), phosphate (P), and silicon (Si) uptake in *Karenia* (at the bloom) and *Chaetoceros* (at the after-bloom) when one of the two species was dominant. For each gene family, the summed gene expression (TPM) is displayed. Asterisks mark upregulated genes in the bloom stage relative to nonbloom or after-bloom in *Karenia*, or upregulated genes in the after-bloom stage relative to the bloom or nonbloom sample groups in *Chaetoceros* (*n* = 3). (B) Phagotrophy and related pathways in *Strombidinopsis*. (C) Top 50 highly expressed viral genes. The viruses in red font represent algae-infecting viruses. (D) Expression of highly expressed microbial genes (TPM, >100) related to cell wall lysis. Larger inner white circles mark higher expression levels of genes. The outer colorful circles represent the gene expression contribution of each species.

To explore what molecular factors drove the transition from the *Karenia* bloom to *Chaetoceros* sp. dominance in the after-bloom stage, we identified HEGs when one of the two species was dominant and DEGs across three sample groups related to N, P, and Si uptake in *Karenia* and *Chaetoceros* species. *Chaetoceros* ammonium transporters were the most highly expressed N-related HEGs when the species was dominant (the after-bloom), followed by amidase, with both being upregulated in the after-bloom samples compared to the bloom or nonbloom samples, while nitrate-nitrite transporter genes and nitrate-nitrite reductase genes were the top two most highly expressed N-related genes in *Karenia* during its bloom (Fig. 6A). Furthermore, a diversity of dissolved organic nitrogen (DON)-related HEGs were detected in *Karenia* at the bloom stage but not in *Chaetoceros* during after-bloom, including allantoicase, allantoinase, amidase, urate oxidase, and urease (Fig. 6A). For P-related genes, both *Chaetoceros* and *Karenia* phosphate transporter and pyrophosphatase genes were not only among the top two highly expressed but also were upregulated (HEGs and DEGs) under their respective dominance conditions. *Karenia* also showed high expression of diphosphatase and 5′-nucleotidase in the bloom sample. In addition, *Chaetoceros* silicon transporters were more highly expressed and upregulated in the after-bloom condition than in the bloom and nonbloom conditions (Fig. 6A). These findings indicated different nutrient uptake strategies between *Karenia* and *Chaetoceros*, which were potentially responsible for the transition from the dinoflagellate bloom to the diatom dominance.

### Transcriptomic signals of active grazing and microbial attacks in nonbloom and after-bloom stages.

The upregulated defense genes in the nonbloom and after-bloom samples prompted us to interrogate whether metabolic pathways related to grazing and microbial attack were correspondingly upregulated. We first examined genes controlling transport and grazing activities in the ciliate *Strombidinopsis*, including genes in the lysosome, phagosome, autophagy, endocytosis, and mitophagy pathways. Results showed that these pathways were indeed expressed at much higher levels in the nonbloom and after-bloom than in the bloom stage (Fig. 6B), involving 261 genes related to endocytosis, phagocytosis, or phagosome (Table S6). These included highly expressed genes encoding 1-phosphatidylinositol-4-phosphate 5-kinase (PIP5K), ADP-ribosylation factor 1 (ARF1), cathepsin L, actin beta/gamma 1, cofilin (CFL), and protein transport protein SEC61 subunit alpha (SEC61A).

We also explored whether viral infection was involved in bloom prevention and collapse and indeed detected viral mRNA in our metatranscriptomic data. The top 50 most highly expressed viral genes belonged to 33 functional types. Twenty-three of these, including four algal viruses (annotated as organic lake phycodnavirus 1 and 2, *Phaeocystis globose* virus, and Beihai weivirus-like virus 11), were most highly expressed in the nonbloom samples. Ten of these showed the highest expression in the after-bloom stage, including Beihai picorna-like virus, major capsid protein-containing viruses, *Chrysochromulina ericina* virus, and *Edafosvirus* sp., four types of algal viruses different from those in the nonbloom stage (Fig. 6C).

Bacteria are another potential bloom killer. Highly expressed genes (TPM, >100 in at least one sample) from prokaryotes that encode enzymes potentially involved in algal cell wall lysis were detected, including genes with expression (listed from high to low expression): aminopeptidase, metalloprotease, sulfatase, xylanase, glucosidase, lysozyme, lipase, chitinase, trypsin, and amylase (Fig. 6D). These 10 bacterial enzymes showed the highest expression in the nonbloom samples, followed by the after-bloom samples. Aminopeptidase, metalloprotease, glucosidase, and trypsin were mainly contributed by Rhohdobacteraceae and unassigned bacteria. Sulfatase, xylanase, lysozyme, and amylase were mainly contributed by Flavobacteriaceae. Lipase was dominantly contributed by Alteromonadaceae. Chitinase was dominantly from Crocinitomicaceae. The cytolytic capacities of bacteria likely contributed to the convergent *Karenia* cell-removing activities, which led to the prevention of bloom formation in the nonbloom stage and the destruction of the bloom in the after-bloom stage. However, the current data did not allow us to exclude the possibility that the attack of grazers (ciliates) and viruses might have led to the release of undigested algal remnants in the nonbloom or after-bloom samples, and bacteria might have used the same enzymes (as reported above) to utilize this rich food source and support their growth.

## DISCUSSION

Multiple processes regulate the dynamics of HABs, including μ, I, Ex, G, and M. Determining which of these most critically influences a specific bloom is crucial for understanding and managing HABs but is a daunting challenge because it requires simultaneous *in situ* measurements of these terms. However, integrative ecological and molecular analyses provide a possibility to approach the problem. Although transcriptomes are indirect indications of *in situ* physiological and ecological dynamics, they are still powerful evidence of these processes. By taking the molecular ecological approach, clues and insights about the most important bottom-up (E, N, D, S) and the top-down (G, M) factors can be interrogated (29), where a strong signal of D in the bloom species corresponds to high activity of G from grazers and/or M from microbes. In the present study, we conducted a study on a *K. longicanalis* bloom using this approach. Based on a shipboard field campaign, triplicated samples were collected from nonbloom, bloom, and after-bloom assemblages and processed immediately on site to capture the *in situ* ecological status and species-specific metabolic profiles. The resulting metatranscriptomes provided the first functional gene catalog of *K. longicanalis*, enriching the existing dinoflagellate genomic resource. The data set also revealed the major basic nuclear protein (MBNP) and growth- and development-associated EF-hand domain-containing protein (48, 49) as highly expressed genes in the bloom-causing dinoflagellate. MBNP in dinoflagellates is located at the periphery of the permanently condensed chromosomes and the nucleolar organizing region, and although its role is unclear (50), it has been consistently reported as one of the most highly expressed genes in dinoflagellates (51), signaling its functional importance. In addition, we simultaneously documented eukaryotic and prokaryotic DNA barcodes (SSU) and their mRNAs under different bloom conditions. With both eukaryotic and prokaryotic plankton covered, the metabarcoding and metatranscriptomic data sets represent the most comprehensive data sets for a bloom study to date, and it enabled a deep inquiry into the complex drivers that regulated bloom growth and demise.

### Strong energy and nutrient acquisition as a major driver of the *K. longicanalis* bloom.

Despite the overall stable transcriptional expression of ENDS genes, probably because most dinoflagellate genes are posttranscriptionally regulated (51), a few genes were remarkably regulated in our transcriptomic data set. The acquisition of energy and nutrients plays a fundamental role in algal growth to form and maintain an algal bloom (52, 53). Under bloom conditions, *Prorocentrum shikokuense* (formerly *P. donghaiense*) is advantaged in energy acquisition with high expression levels of genes encoding proton pump rhodopsin, nutrient transporters, and enzymes involved in the utilization of organophosphates (27, 29). The present study found that many genes related to energy acquisition, including photosynthesis and carbon fixation, were significantly upregulated under the bloom condition, generally in congruence with the bloom of *P. shikokuense* (27, 29). The HEGs were uniquely enriched in the fatty acid degradation pathway, suggesting active catabolism of fatty acids to generate energy to support bloom growth. Furthermore, the ATP synthase gene of *Karenia* was also remarkably upregulated under the bloom condition. All these suggest energy acquisition was active during the bloom.

The strongest expression of nitrate-nitrite transporters and reductase genes represented in the N-related HEGs is suggestive of active utilization by the abundant cells of the bloom species. Indeed, the ambient dissolved inorganic nitrogen (DIN; NH_4_^+^, NO_2_^−^, and NO_3_^−^) was depleted under the bloom condition (<5 μM) relative to the nonbloom and after-bloom conditions (Fig. 2A), evidently as a result of rapid consumption. Despite the DIN depletion, DON was copious (>15 μM) during the bloom, probably derived from unconsumed aquaculture feed in the intense aquaculture operation (54). In support of this proposed source of DON, we found that DOC was also most abundant at the bloom stage. Likely, this source of DON played an important role in supporting *Karenia* bloom growth, because our metatranscriptomic data indicated that *Karenia* was actively scavenging DON during the bloom. These bloom-active DON genes included urate oxidase, which catalyzes the oxidative decarboxylation of uric acid to allantoin, which is essential in nitrogen assimilation in many plants (55). Allantoicase, functioning in ureide catabolism to generate a nitrogen source (56), was found to be inducible in the green alga Chlamydomonas reinhardtii when taking up ureide (57). Allantoinase, an enzyme that catalyzes the conversion of allantoate into ureidoglycolate and urea (58), was shown to increase in C. reinhardtii when it was using allantoin (59). Urease catalyzes urea degradation to NH_4_^+^ and CO_2_ to support the growth of many algae on urea as the nitrogen source (60). In addition, amidase, which catalyzes the hydrolysis of amides to ammonia and free carboxylic acids (61), was also highly expressed in *K. longicanalis* during the bloom. Moreover, there was strong and elevated expression of amino acid transporters, suggesting the potential that *K. longicanalis* was actively utilizing amino acids during the bloom. In addition, the HEGs found under the bloom condition were enriched in the endocytosis pathway, suggesting potential involvement of mixotrophy in nutrient acquisition for bloom development.

Similar to N nutrition, the high expression and/or upregulation of phosphate transporter and phosphatases in the bloom stage suggest active inorganic P uptake and organic P scavenging in *Karenia* under bloom conditions. Among the phosphatases highly expressed in *K. longicanalis* were pyrophosphatase (upregulated), diphosphatase, and 5′-nucleotidase (5NT). Pyrophosphatase, which hydrolyzes the high-energy pyrophosphate into two inorganic phosphate molecules (62), has been reported in the dinoflagellate Alexandrium tamarense to hydrolyze phosphodiesters for P nutrient (63). Diphosphatase catalyzes diphosphate into phosphates (64). 5NT has been linked to ATP utilization in *K. mikimotoi* (65). All the N and P nutrition results indicated that the bloom species was exploiting all sources of nutrients, including DIN, DON, DIP, DOP, and possibly phagotrophy during the bloom.

Active energy and nutrient acquisition is expected to promote cell proliferation via sexual and asexual reproduction. Previous molecular studies on dinoflagellate blooms have also revealed increases in metabolic activities of energy production, carbon metabolism, transport, and synthesis of cellular membrane components (an indicator of cell division) as potential drivers of blooms (23, 24, 26, 28). As expected, we detected significantly elevated expression of genes coding for cell division proteins. As none of the canonical meiosis core genes was found upregulated in the bloom reported here, contrary to the *K. mikimotoi* and other dinoflagellate blooms (25), the role of sexual reproduction in this *K. longicanalis* bloom was likely weak, although it is not impossible that a significant role might have escaped our detection. Nevertheless, our data provide strong evidence that energy and nutrient acquisition was the major metabolic driver of the bloom of *K. longicanalis*.

### Microbial attack, ciliate grazing, and diatom competition linked to *K. longicanalis* bloom prevention and termination.

Microbial attacks and zooplankton grazing are two major top-down controls of a phytoplankton bloom that can prevent or terminate a bloom, and for a bloom to occur the species needs to have strong defense against these cell-damaging and removing processes. The higher expression of defense mechanisms in *K. longicanalis* in the nonbloom sample group suggests that a more vigorous microbial attack might have diverted intracellular resources to defense at the cost of cell proliferation. Conversely, the considerable decreases in diversity and activity of viral genes under the bloom conditions suggest a lower infection activity during the bloom. Several viruses were transcriptionally very active under nonbloom or after-bloom conditions. Picorna-like viruses are positive-sense single-stranded RNA viruses that are major pathogens of plants, animals, insects, and marine phytoplankton (66, 67). They are widely distributed in marine water (68, 69) and have been reported to infect the bloom species Heterosigma akashiwo (66, 70). Mimivirus edafos virus is a type of nucleo-cytoplasmic large DNA virus (NCDLV) (71) that can infect diverse eukaryotes, such as protists and algae (72, 73). The *TARA Oceans* expedition data showed that NCDLVs outnumbered eukaryotic organisms in the photic layer (74). *Chrysochromulina ericina* viruses and phycodnaviruses are algae-infecting large DNA viruses widely distributed in aquatic environments (75–77). *Phaecystis globaosa* virus (78), Beihai weivirus-like virus 11 (79), and many major capsid protein-containing viruses have been reported to be microalga-infecting viruses (80, 81). With the highest transcriptional activities under the after-bloom or nonbloom condition, picorna-like viruses, edafos virus, and other detected algal viruses might play an important role in *K. longicanalis’* decline. Further laboratory experiments are needed to demonstrate the infection of these viruses in *K. longicanalis*.

Bacterial genes encoding cytolytic enzymes were also actively expressed in the nonbloom and after-bloom stages (Fig. 6D), suggesting their potential degradation of *K. longicanalis* cell walls. These enzymes have been reported to function in lysing or inhibiting algal cells. Extracellular enzymes, including aminopeptidase and lipase produced by *Cytophaga* sp., have been linked to *Alexandrium catenella* cell lysis (82). Chitinase, sulfatase, and trypsin can inhibit the growth of various *Chlorella* strains by digesting the cell walls (83). Amylase from marine bacteria can be used to saccharify marine microalgae (84). Glucosidase and lysozyme can hydrolyze the cell walls of *C. vulgaris* (85). Xylanase (86, 87) and metalloproteases (88) produced by bacteria have tremendous potential to degrade cell walls. Rhodobacteracea was the most abundant bacteria family with the highest gene expression in the nonbloom samples, and its abundance was negatively correlated with the abundance of *K. longicanalis*. Coincidentally, a marine Rhodobacteraceae bacterium strain has been reported to exact an 87% inhibitory rate on *K. mikimotoi* (89), a sister species of *K. longicanalis*. The highest expression of aminopeptidase, metalloprotease, glucosidase, and trypsin in Rhodobacteracea bacteria further supported the bacteria’s role in inhibiting *K. longicanalis* growth. In addition, Flavobacteriaceae and *Vibrionaceae* both had the highest gene expression activities in the nonbloom samples. Many Flavobacteriaceae bacteria have been reported to have algicidal activity against algae, including *K. mikimotoi* (90, 91) and *K. brevis* (92). The highest expression of sulfatase, xylanase, lysozyme, and amylase in Flavobacteriaceae in the nonbloom samples provided strong evidence for the ability of Flavobacteriaceae to disrupt algal cell walls. Vibrionaceae bacteria, especially *Vibrio*, have been reported to have algicidal activity for *K. mikimotoi* (93), *A. tamarense* (94), and *Akashiwo sanguinea* (95). This bacterial lineage probably also played an active role in inhibiting the growth of *K. longicanalis*. All these microbes showed the highest cell wall lysis potential under the nonbloom or after-bloom conditions, suggesting their potential roles in influencing the bloom’s initiation and termination. However, in the natural plankton assemblage, the microbial interactive network might be much more complex. For instance, the increase of bacteria and bacterial hydrolysis in the nonbloom and after-bloom stages might have been a coincidence resulting from a microbial trophic network where ciliate slopping grazing and viral cytolytic activities produced organic matter that fueled bacterial growth. Thus, further investigation is needed in the future to directly attribute the bacterial activities to the bloom suppression and termination.

In marine ecosystems, ciliates and other heterotrophic protists are the major phytoplankton consumers (96) and strongly affect phytoplankton population dynamics (97); they regulate harmful algal bloom development as a top-down control (98–100). It was previously reported that *Strombidinopsis* sp. (ca. 150 μm in cell length) could feed on many species of dinoflagellates (101–104). In the present study, endocytosis- and phagosome-related genes were more highly expressed in *Strombidinopsis* in the nonbloom and after-bloom sample groups, indicating that grazing of *K. longicanalis* by *Strombidniopsis* might have prevented bloom formation or accelerated the bloom decline in the nonbloom area or period. For instance, PIP5K modulates the actin cytoskeleton during the attachment and ingestion phases of phagocytosis (105). CFL is involved in the formation and disruption of the phagocytic cup (106). ARF are key proteins of endocytosis in plants (107). The expression levels of these genes and the relative abundance of *Strombidinopsis* were the highest in the nonbloom samples but were also high in the after-bloom samples.

Finally, competition by coexisting phytoplankton can also shape the dynamics of a phytoplankton species. Upon the decline of the *K*. *longicanalis* bloom under the after-bloom condition, the diatom *Chaetoceros* sp. grew to be dominant in the assemblage. The high and upregulated expression of the diatom silicon transporter in the after-bloom stage compared to the bloom stage was evidence that *Chaetoceros* grew more quickly in the after-bloom stage and hence had a higher demand for nutrients. For N-nutrient uptake, *Chaetoceros* showed the highest expression of ammonium transporter, consistent with the increased NH_4_^+^ concentration that likely resulted from bloom decline in the after-bloom stage, indicating that NH_4_^+^ might contribute to the growth of this diatom. The high and elevated expression of phosphate transporter and pyrophosphatase in *Chaetoceros* during after-bloom was also evidence of rapid DIP and DOP uptake, imposing competition with *K. longicanalis*. This nutrient competition by the diatom might have contributed to the termination of the dinoflagellate bloom.

While the high activities of ciliates, bacteria, and viruses likely substantially contributed to the prevention and termination of the *K. longicanalis* bloom, it is important to note that other factors might have been at play in the natural marine environment. The data reported here only represent snapshots of the bloom emergence and demise processes and might have missed some changes in the environment, other activities of the coexisting organisms, or possible vertical migration of the bloom-causing dinoflagellate. Future studies need to investigate those other potential factors with simultaneous cell-level physiological measurements with more intense time- and space (vertical)-resolved sampling.

### Conclusions.

This study provides the first transcriptome of *K. longicanalis* and represents the first natural bloom investigation of this species. Acquisition of energy and nutrients, defense, cell proliferation, and cell mortality were explored as potential regulators of a *Karenia longicanalis* bloom using an integrated molecular ecological approach. Our integrative data analysis indicated that the promoted cell proliferation and bloom formation in the bloom stage were supported by high or increased expression of energy and nutrient acquisition and metabolism genes in *Karenia.* Cell mortality due to microbial attack and ciliate grazing appeared to be the major inhibitor of bloom formation in the nonbloom stage and the promoter of bloom demise in the after-bloom stage. The competition of the *Chaetoceros* sp. likely also contributed to the decline of the *K. longicanalis* population and the rise of the *Chaetoceros* sp. population to dominance in the after-bloom stage. Our metatranscriptomic data suggested that *K. longicanalis* was heavily “invested” in defense during the nonbloom or after-bloom stages, but defense was inadequate to overcome the overwhelming grazing and microbial attacks. Therefore, mechanisms underlying the *K. longicanalis* bloom appear to be different from those regulating the bloom of *P. shikokuense*, which requires the upregulation of genes related to energy and nutrient acquisition, defense, and sexual reproduction (25, 27, 29), or of *K. mikimotoi*, for which sexual reproduction appears to be influential (25). Of the four elements in ENDS, the most notable bloom-promoting factors for the *K. longicanalis* bloom were E and N, with the contribution of D and S being apparently weak, and the biotic bloom-inhibiting or -dissipating factors were evidently the failure of *K. longicanalis* to defend against microbial attack and grazing and to outcompete diatoms for nutrients. This novel insight into HAB-regulating mechanisms, along with the first transcriptomic data for *K. longicanalis* and the integrative molecular ecological approach, will prove to be a valuable resource and essential foundation for further elucidation of bloom regulators of this and related species of Kareniaceae in the future.

## MATERIALS AND METHODS

### Sample collection and environmental factor measurements.

Three sampling events, each in triple biological replicates, were launched to represent a nonbloom condition (nonbloom 1 to 3), a bloom condition (bloom 1 to 3), and an after-bloom condition (after-bloom 1 to 3) (Fig. 1A). Fifty-milliliter seawater samples were collected and fixed in Lugol's solution (2%) for subsequent microscopic examination to identify species and enumerate cell concentrations in a Sedgwick-Rafter counting chamber. The bloom condition was sampled on June 11 when the cell concentration of the bloom species was 1.51 × 10^7^ cells/liter. The nonbloom assemblage was sampled on the same day but from outside the bloom patch within the bay, where the cell concentration of the bloom species was 600-fold lower (2.4 × 10^4^ cells/liter). The after-bloom condition was sampled in the same location as the previously sampled bloom area on June 12, when the abundance of the bloom species declined by 137-fold (1.1 × 10^5^ cells/liter). At each sampling event, three separate samples were collected as replicates, giving a total of 9 samples. For each sample, 20 liters of water was taken from the subsurface (~0.5 m) and prescreened through a 200-μm mesh to exclude large zooplankton. Immediately afterward, the sample was filtered onto 3-μm polycarbonate filters (47 mm). Filtration was completed in 15 min to minimize changes in gene expression. Each filter was split into four equal parts using clean scissors, one for DNA analysis and the other three for RNA analysis. Cells retained on the filter membranes included planktonic eukaryotes (3 to 200 μm) and microbes that were infecting or attached onto the eukaryotic cells. Filters for RNA sequencing were immediately immersed in 1 mL TRIzol in a 2-mL tube and kept frozen in liquid N_2_ during the cruise and, upon return to the laboratory, were stored at −80°C until RNA extraction. Samples for DNA sequencing were transferred to a 2-mL tube containing 1 mL DNA lysis buffer (10 mM Tris-HCl [pH 8.0], 100 mM EDTA [pH 8.0], 0.5% SDS, 200 μg mL^−1^ proteinase K) and held at −20°C until DNA extraction.

A comprehensive set of environmental factors was also measured. Seawater temperature, dissolved oxygen, conductivity, pH, and salinity were measured *in situ* using YSI Professional Plus, and surface light was measured using a digital lux meter. Two sets of 50-mL seawater samples from each of the three sampling events were filtered onto GF/F membranes and a 0.2-μm membrane and stored at −80°C for chlorophyll *a* and nutrient measurements, respectively. Chlorophyll *a* was measured using a Trilogy laboratory fluorometer (Turner Designs, USA). The concentrations of N nutrients (NH_4_^+^, NO_3_^−^, NO_2_^−^), PO_4_^3−^, and SiO_4_^4−^ were measured using the Bran-Lubbe AAIII system (Germany) on water samples that had been filtered through a 0.22-μm membrane and kept at −80°C. DOC and total dissolved nitrogen (TDN) were measured using the high-temperature catalytic oxidation method with a Multi N/C 3100 system (Germany). DON concentration was estimated based on TDN minus DIN (NH_4_^+^ + NO_3_^−^ + NO_2_^−^). For particulate organic phosphorus and particulate organic nitrogen (108–110), samples were first digested with the high-temperature and high-pressure REDOX methods, and the resulting inorganic products were measured using the Bran-Lubbe AAIII system (Germany).

### DNA extraction and barcoding sequencing.

DNA was extracted as previously reported (111). The DNA was used as the template for PCR amplifications of the 16S rRNA genes and 18S rDNA from prokaryotes and eukaryotes, respectively. The 16S rRNA V4 variable region (~290 bp) was amplified with the 515F-806R bacterial/archaeal primer pair 515F (5′-GTGCCAGCMGCCGCGGTAA-3′) and 806R (5′-GGACTACHVGGGTWTCTA AT-3′) (112). The 18S variable region (V4, 450 bp) was amplified with primers 18S V4-F (5′-GGCAAGTCTGGTGCCAG-3′) and 18SV4-R (5′-GACTACGACGGTATCTRATCRTCTTCG-3′) (113). The 28S RNA gene was amplified with primers LSU rDNA-D1R-F (5′-ACCCGCTGAATTTAAGCATA-3′) and LSU rDNA-D2C-R (5′-CCTTGGTCCGTGTTTCAAGA-3′). The PCR products were then sequenced. The 16S rRNA gene amplicons were sequenced on the Hiseq2500 platform (25,000 2 × 250-bp read pairs). The 18S rDNA amplicons were sequenced on the Miseq platform (25,000 2 × 300-bp read pairs). The 28S rRNA gene was amplified using nested PCR with primers KI_28sF (5′-TAAGCGGAGGATAAGAAACTAAATAGG-3), KI_28sR1 (5′-CCGTGTTTCAAGACGGGTCGAATAA-3′), and KI_28sR2 (5′-AACCATTTCGTCATCGTACTTATGTC-3′).

### RNA extraction and sequencing.

RNA extraction was performed as previously reported (27, 29) with minor modifications. Briefly, cells were rinsed off the filter with TRIzol using a pipette. The cell suspension was mixed with beads (Biospec, USA) and shaken at 6 m/s on a FastPrep-24 bead mill (MP Biomedicals, USA) two or three times to ensure complete cell breakage. The cell lysate was then subjected to RNA extraction following protocols of the TRI reagent and the Direct-zol RNA columns (114). One microgram of RNA with the RNA integrity number (RIN) above seven was used for library preparation according to the manufacturer’s protocol (NEBNext Ultra directional RNA library prep kit for Illumina). To yield both prokaryotic and eukaryotic transcriptomes, instead of using oligo(dT)-based mRNA isolation, we removed rRNA from total RNA using the Ribo-Zero rRNA removal kit (bacteria; Illumina). cDNA libraries were prepared from the resulting rRNA-depleted RNA and sequenced following Illumina Hiseq PE150 protocols.

### Bioinformatics analyses.

**(i) Community classification and taxon interactions.** The raw data from the 16S and 18S amplicon sequencing were quality filtered to eliminate the adapters and low-quality reads to obtain clean reads with TrimmomaticPE. The parameters were set as follows: leading, 5; trailing, 5; sliding window, 4:15; minlen, 50. The clean data were used for feature identification and quantification using the UPRASE with NOISE3 method (115). Taxonomic assignment was carried out using SILVA 138.1 and the NCBI nucleotide database. The taxon-taxon and taxon-environment correlations were analyzed and visualized using ggcor packages in R software (116). Correlations between taxa and environmental factors were analyzed using the Pearson model, and those between species were analyzed using the Mantel test. Those with *P* values of <0.05 were considered significantly correlated.

**(ii) Metatranscriptomic read assembly.** Sequencing reads from each RNA sample were assembled with MEGAHIT v1.0 (117). The assembled unigenes from the triplicate samples of each bloom condition (group) were then clustered using CD-HIT (v4.6.5) (118) with the default parameters of “-d 0 -c 0.95 -G 0 -aL 0.95 -AL 100 -aS0.95 -AS 30” and TGICL (v4.6.5) (119). Finally, the unigenes from the three groups were merged into the final nonredundant reference metatranscriptome using the CD-HIT and TGICL software.

**(iii) Gene taxonomic assignment and functional annotation.** To identify the species origin of the unigenes in the reference metatranscriptome, a comprehensive in-house database (nrMegPhylodb) (120) was created by integrating several existing databases. These included protein sequences of the NCBI nonredundant protein database (NR, June 2019), PhyloDB (version 1.076), and proteomes of four dinoflagellates from our laboratory, including *P. shikokuense*, *K. mikimotoi*, *Effrenium* sp., and Karlodinium veneficum. BLASTX was used to match our reference metatranscriptome against the in-house database with an E value of 1E−3, and the taxonomic source of the best-hit gene was assigned to the query unigene sequence.

Functional annotation of the unigenes in the reference metatranscriptome was conducted using Diamond (121), based on a Blastx search against such databases nrMegPhylodb, eggNOG (v4.5), KEGG (April 2018), and Swiss-Prot (October 2018). Gene ontology was assigned based on NR annotation.

**(iv) Gene expression quantification and differential expression analysis.** The raw reads of the metatranscriptomes were trimmed and quality-filtered using Cutadapt (v1.9.1) with parameters setting at “-m 75 –max-n 0.1 –discard-trimmed -q 20.” Clean reads from each sample were mapped to the above-described reference metatranscriptome using the aligner Bowtie2 and quantified using RSEM (v1.2.4) with default parameters. Gene expression at the community level (community TPM) was calculated as transcripts of the specific gene per million of total mapped reads of the community and used to compare gene expression contributions of different lineages or kingdoms to the whole community. While comparing gene expression differences in the same species between sample groups, gene expression at the species level was renormalized as transcripts of the specific gene from the specific species per million total mapped reads of the species (species TPM) was used. Only genes with an average gene expression TPM of ≥0.1 were used in differential expression analyses within species, including *K. longicanalis* and *Chaetoceros* sp., for which the DESeq package in R software was employed. DEGs were defined as those with an adjusted *P* value of <0.05 and fold change of ≥2. DEGs associated with energy and nutrient acquisition, defense, and cell reproduction were identified using a *t* test based on average gene expression (TPM) of the gene family, as previously reported (25).

### Data availability.

Raw sequence reads of mRNA and the DNA metabarcodes were deposited in the NCBI Sequence Read Archive (BioProject ID PRJNA689700).
