# Supplementary material for: In Situ Molecular Ecological Analyses Illuminate Distinct Factors Regulating Formation and Demise of a Harmful Dinoflagellate Bloom
Source: Microbiol Spectr. 2023 Apr 19;11(3):e05157-22. doi: 10.1128/spectrum.05157-22 (PMC10269597; doi:10.1128/spectrum.05157-22)
Supplement: Supplemental file 1 — Supplemental material. Download spectrum.05157-22-s0001.pdf, PDF file, 0.3 MB [file spectrum.05157-22-s0001.pdf]

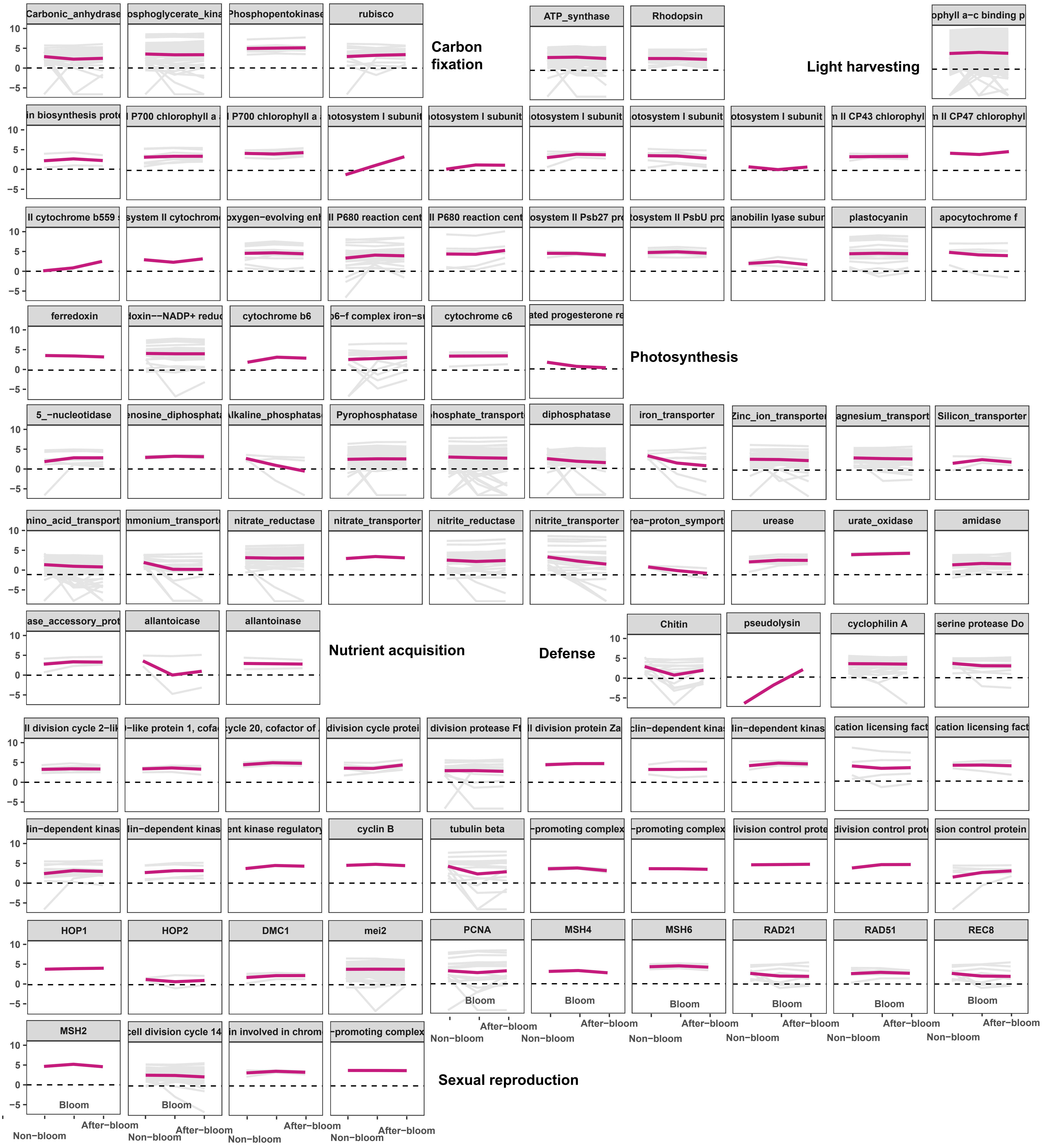


**Fig. S1.** Expression patterns of energy and nutrient acquisition, defense, and cell reproduction (ENDS) related genes in Karenia under the three bloom conditions (non-bloom, bloom and after-bloom).
